# Integrating Insect Life History and Food Plant Phenology: Flexible Maternal Choice Is Adaptive

**DOI:** 10.3390/ijms17081263

**Published:** 2016-08-03

**Authors:** Minghui Fei, Jeffrey A. Harvey, Berhane T. Weldegergis, Tzeyi Huang, Kimmy Reijngoudt, Louise M. Vet, Rieta Gols

**Affiliations:** 1 Department of Terrestrial Ecology, Netherlands Institute of Ecology, Droevendaalsesteeg 10, 6708 PB Wageningen, The Netherlands; m.fei@nioo.knaw.nl (M.F.); j.harvey@nioo.knaw.nl (J.A.H.); l.vet@nioo.knaw.nl (L.M.V.); 2 Section Animal Ecology, Department of Ecological Sciences, VU University Amsterdam, De Boelelaan 1085, 1081 HV Amsterdam, The Netherlands; 3 Laboratory of Entomology, Wageningen University, Droevendaalsesteeg 1, 6708 PB Wageningen, The Netherlands; weldegergis@gmail.com (B.T.W.); tzeyipastry@gmail.com (T.H.); kimmy.reijngoudt@wur.nl (K.R.)

**Keywords:** endoparasitoid, foraging, glucosinolate, herbivore, herbivore induced plant volatile (HIPV), multivoltine, oviposition, rearing history, plant volatiles

## Abstract

Experience of insect herbivores and their natural enemies in the natal habitat is considered to affect their likelihood of accepting a similar habitat or plant/host during dispersal. Growing phenology of food plants and the number of generations in the insects further determines lability of insect behavioural responses at eclosion. We studied the effect of rearing history on oviposition preference in a multivoltine herbivore (*Pieris brassicae*), and foraging behaviour in the endoparasitoid wasp (*Cotesia glomerata*) a specialist enemy of *P. brassicae*. Different generations of the insects are obligatorily associated with different plants in the Brassicaceae, e.g., *Brassica rapa*, *Brassica nigra* and *Sinapis arvensis*, exhibiting different seasonal phenologies in The Netherlands. Food plant preference of adults was examined when the insects had been reared on each of the three plant species for one generation. Rearing history only marginally affected oviposition preference of *P. brassicae* butterflies, but they never preferred the plant on which they had been reared. *C. glomerata* had a clear preference for host-infested *B. rapa* plants, irrespective of rearing history. Higher levels of the glucosinolate breakdown product 3-butenyl isothiocyanate in the headspace of *B. rapa* plants could explain enhanced attractiveness. Our results reveal the potential importance of flexible plant choice for female multivoltine insects in nature.

## 1. Introduction

Herbivorous insects have to locate their host food plants often embedded in patches that may be species-rich and structurally and chemically complex [1,2]. Their co-evolved natural enemies, such as parasitoids and predators, are similarly challenged when they are searching for hosts or prey [1,2]. Location of these resources during foraging is often characterized by a gradual narrowing down of the area in which these resources can be found, and is described as a reliability-detectability problem [3]. For example, insect herbivores first have to find the proper habitat and then locate a suitable food plant within this habitat; their parasitoids must also overcome these same challenges to locate hosts that are often small and feeding in concealed locations on the food plant. Once potential food plants and/or hosts have been located, these can be accepted as oviposition sites or rejected, which is largely determined by differences in the suitability and quality of the resources for their development [4,5]. The first steps, i.e., habitat and host location, of this sequential process eventually leading to successful insect development primarily rely on visual and olfactory cues utilized by the insects [6,7,8]. In particular, volatiles that are released by plants in response to herbivore feeding (so-called herbivore induced plant volatiles (HIPVs)) have been extensively studied in relation to parasitoid foraging behaviour over the past 25 years [7,9,10,11,12,13].

The vast majority of insect herbivores are specialists that feed on only a few related plant species in nature [14,15]. Therefore, an herbivore and its specialist parasitoids are expected to rely on specific cues that are related to their food plants or hosts [7,16,17] such as phylogenetically conserved secondary (defensive) metabolites [18]. For example, larvae of the cabbage butterflies *Pieris brassicae* and *P. rapae*, primarily feed on plant species in the family Brassicaceae that produce inducible glycoside compounds called glucosinolates [19,20]. Gravid female butterflies of these two species use these glucosinolates to recognize suitable food plants for their offspring by using their tarsi to “scratch” plant tissues prior to oviposition [17,21].

Once they are in the appropriate habitat containing suitable plants or hosts, learning and subsequent experience may further influence the foraging behaviour of insect herbivores and their natural enemies when they are searching for resources. However, the strength of this effect often depends on the developmental stage at which the experience occurs. For example, oviposition experience of adult female parasitoids in the presence of characteristic volatile blends often enhances the parasitoid’s response to these volatiles when these are offered in the absence of hosts [22]. This behavioural adaptation is referred to as associative learning and has been observed in both insect herbivores and their natural enemies [23]. Natal experience, which is obtained during larval feeding and growth, may also affect habitat preferences later in life during the adult stage. Known as the Hopkins’s host selection principle (HHSP), it is, however, controversial as it implies that some form of imprinting is maintained during metamorphosis affecting later developmental stages [24] and also because evidence supporting the principle is thus far scarce [25]. Another major problem with the HHSP is that it does not take into account constraints imposed by temporal changes in diet that may be predicated by life history characteristics of the consumer and its resource. For insects that must switch plant diets from one generation to another (e.g., where the progeny exploit a different species of plant from their parents), it is clear that larval imprinting on a plant may be maladaptive if it hinders the ability of the insects to find and locate new resources that are chemically different from those on which they developed. Thus far, however, most studies tacitly assume that specialist herbivores exploit the same food plant species over many generations, making natal imprinting adaptive.

Some studies have shown that pre-adult experience can affect later foraging behaviour in insects for oviposition sites, at least if these sites are the same or at least very similar to those on which the offspring developed [26,27]. Furthermore, when natal experience influences later habitat choice, it increases the acceptance of the natal habitat type [28]. Preference for the natal habitat type could be beneficial for insects, because natal experience can influence plastic traits, such as the response to cues used during foraging, which make them better adapted to exploit the same resources in similar habitats [29]. Such preference is adaptive because it reduces the costs associated with exploring multiple habitats and in assessing the suitability of these habitats [27]. However, the strength of adaptation also depends on the degree to which the environment changes across space and time in relation to the generation time of the insects.

Many species of herbivorous insects are multivoltine and thus have two or more generations per year [30]. Moreover, some of these herbivores are known to feed on short-lived annual plants that are present in the field for only two or three months during the growing season [31]. Under these conditions, successive generations of herbivores that rely on short-lived annuals for food are obligated to leave the natal plant patch to locate and oviposit on a different plant species that may be different from the plant on which they developed and which grows a considerable distance (kilometers) away. Specialist multivoltine parasitoids of these herbivores are faced with the same constraints related to habitat and host location and thus must track them from one habitat patch to another.

In this study, we investigate the effect of rearing history on oviposition preference for different related host plant species in a multivoltine herbivore *Pieris brassicae* L. (Lepidoptera: Pieridae) and host plant preference behaviour in its endoparasitoid *Cotesia glomerata* L. (Hymenoptera: Braconidae). Caterpillars of *P. brassicae* are specialized on brassicaceous plant species of which all native species over much of its range are short-lived annuals. In the Netherlands, the species has generally three generations per year depending on temperature. The three annual plants studied here, wild turnip, *Brassica rapa* L., charlock mustard, *Sinapis arvensis* L., and black mustard, *Brassica nigra* L., were grown in temporal sequence and are important wild food plants for successive generations of *P. brassicae* in The Netherlands [31]. These plant species tend to grow in dense stands, which is a prerequisite for survival of *P. brassicae* because females lay eggs in clusters that need several plants to sustain their larval development [32]. *Cotesia glomerata* is a specialized gregarious endoparasitoid, i.e., females lay several eggs in the host at a single oviposition event. It primarily attacks early caterpillar stages of *P. brassicae* and it has two to three generations in the Netherlands, also depending on temperature.

The main aim of this study is to determine whether rearing history (i.e., insects reared on the different food-plant species [*B. rapa*, *S. arvensis*, *B. nigra*]) in one generation affects maternal preference of *P. brassicae* and foraging behaviour of *C. glomerata* for the three different plants infested with *P. brassicae* caterpillars. We hypothesize that the rearing history of the two insects will not affect preference for food plant species (herbivore) or volatile-mediated foraging (parasitoid) of future generations because pre-adult conditioning on the natal plant may confer costs e.g., the insects remaining within the natal patch may only encounter plants that are dying and are thus nutritionally unsuitable.

## 2. Results

### 2.1. Host-Plant Oviposition Preference of Pieris brassicae Butterflies

**Figure 1:**
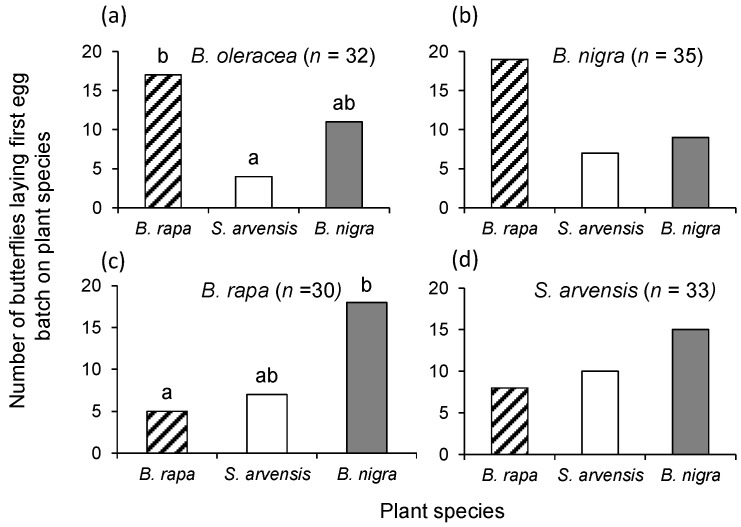
Oviposition preference of female *Pieris brassicae* that had been reared on: *Brassica oleracea* (**a**); *Brassica nigra* (**b**); *Brassica rapa* (**c**); and *Sinapis arvensis* (**d**) in a three-way choice assay with *B. rapa* (**dashed bars**), *S. arvensis* (**white bars**), and *B. nigra* (**grey bars**) plants. Bars represent the total preference number, and bars with the same letter are not significantly different from each other (pairwise χ^2^ test with a Bonferroni correction for multiple comparisons Type I errors). Samples sizes are given in brackets.

There was a significant difference in oviposition preference when the *P. brassicae* butterflies were reared on *B. oleracea* (χ^2_2_^ = 7.94, *p* = 0.02 Figure 1a), *B. nigra* (χ^2_2_^ = 7.09, *p* = 0.03, Figure 1b) or *B. rapa* (χ^2_2_^ = 9.80, *p* = 0.007, Figure 1c), whereas this was not the case for butterflies that were reared on *S. arvensis* (χ^2_2_^ = 2.36, *p* = 0.31, Figure 1d). When reared on *B. oleracea*, female *P. brassicae* butterflies preferred to lay eggs on *B. rapa*, though this preference was only statistically significant for the pair-wise *B. rapa*–*S. arvensis* comparison. When reared on *B. nigra*, *P. brassicae* marginally preferred to lay eggs on *B. rapa* (*B. rapa* vs. *S. arvensis* (α = 0.0167): χ^2_1_^ = 5.54, *p* = 0.019; *B. rapa* vs. *B. nigra*, χ^2_1_^ = 3.57, *p* = 0.059, Figure 1b). With a rearing history on *B. rapa* or *S. arvensis*, butterfly oviposition preference ranked from low to high *B. rapa* < *S. arvensis* < *B. nigra* (Figure 1c,d). Statistically, preference was significant only for the *B. rapa–B. nigra* pair-wise comparison for butterflies reared on *B. rapa*.

### 2.2. Host-Plant Landing Preference of the Parasitoid, Cotesia glomerata

**Figure 2:**
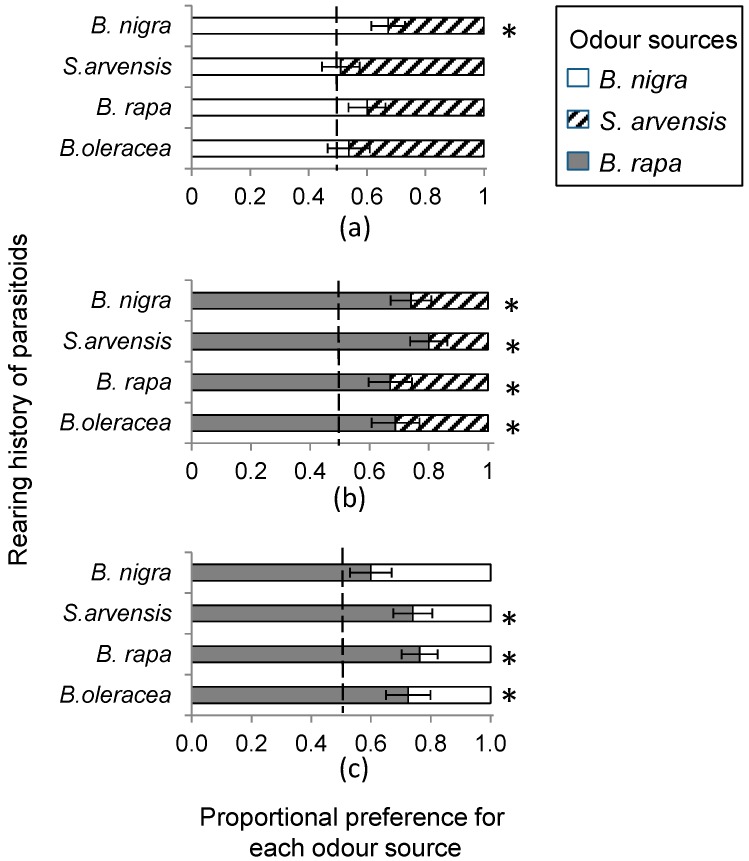
Landing preference of female *Cotesia glomerata* that had been reared on *Brassica oleracea*, *Brassica rapa*, *Sinapis arvensis*, or *Brassica nigra*, in a pair-wise choice assays with: (**a**) *B. nigra* (**white bars**) and *S. arvensis* (**dashed bars**); (**b**) *B. rapa* (**grey bars**) and *S. arvensis*; or (**c**) *B. rapa* and *B. nigra* when infested with *Pierisrs brassicae* caterpillars for 24 h. Bars present the mean proportion (±standard error of the mean or SE) of choice based on 10 replicate bioassays each tested with 10 responding wasps. An asterisk indicates a significant preference within a plant pair.

**Figure 3:**
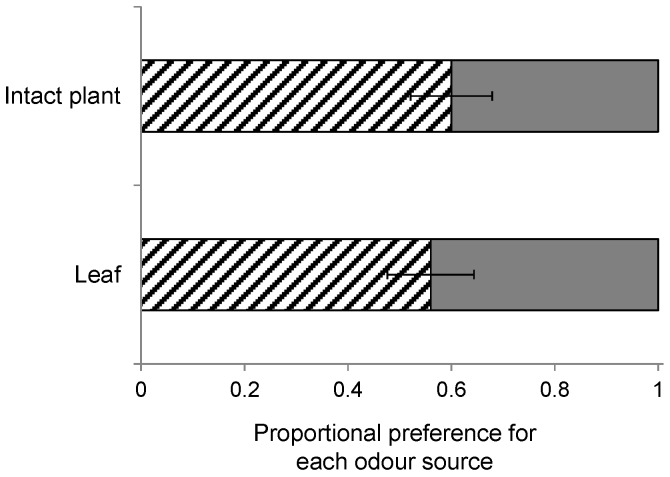
Landing preference of female *Cotesia glomerata* in a choice bioassay with intact plants (**top bar**) or leaves (**bottom bar**). Wasps had been reared on *Brassica nigra* and were given the choice between *Brassica rapa* (**dashed bars**) and *B. nigra* (**grey bars**) infested with 20 first instar *Pieris brassicae* for 24 h. Bars present the mean proportion (±SE) of choice based on 10 replicate bioassays each tested with 10 responding wasps.

**Figure 4:**
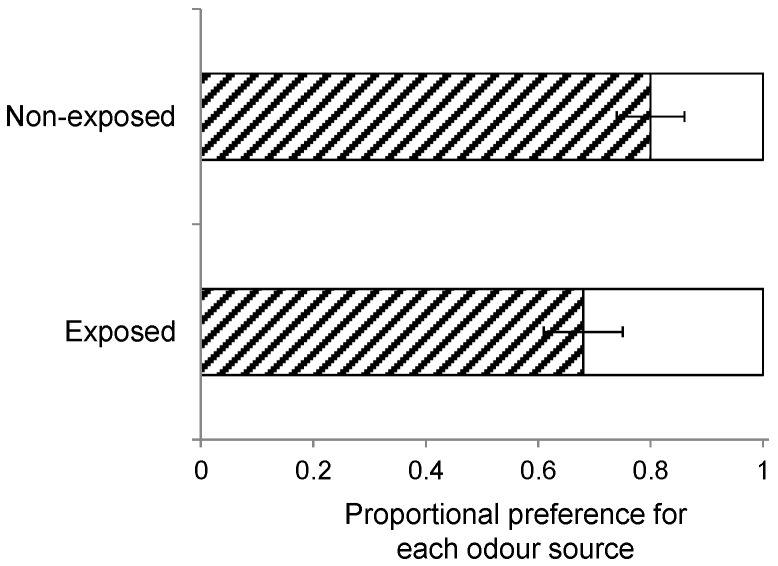
Landing preference of female *Cotesia glomerata* eclosing from cocoons produced by host caterpillars that had been removed from the plants before the parasitoid larvae egressed from the host (non-exposed group, **top bar**) and those eclosing from cocoons that were left on the plants on which the caterpillars had developed until adult eclosion (exposed group, **bottom bar**). Wasps had been reared on *Sinapis arvensis* and were given the choice between a *Brassica rapa* (**dashed bars**) and a *S. arvensis* plant (**white bars**) infested with 20 first instar *Pieris brassicae* for 24 h. Bars present the mean proportion (±SE) of choice based on 10 replicate bioassays each tested with 10 responding wasps.

In total, 1270 wasps made a choice in the wind tunnel, which was 92% of the wasps that were initially released in the wind tunnel. Rearing history had no effect on volatile-mediated foraging behaviour (Figure 2; *B. nigra* vs. *S. arvensis*: χ^2_3_^ = 4.02, *p* = 0.26; *B. rapa* vs. *S. arvensis*: χ^2_3_^ = 2.11, *p* = 0.55; *B. nigra* vs. *B. rapa*: χ^2_3_^ = 3.68, *p* = 0.30 based on generalized linear model (GLM) analyses. Overall, wasps clearly preferred host-infested *B. rapa* plants over *S. arvensis* (*t*_37_ = 7.0, *p* < 0.001) and *B. nigra* plants (*t*_39_ = 6.2, *p* < 0.001), though, in the latter case, this preference was less pronounced when the wasps had been reared on *B. nigra* (Figure 2c). They also preferred host-infested *B. nigra* over *S. arvensis* plants (*t*_38_ = 2.7, *p* = 0.01), especially when they had been reared on *B. nigra* (Figure 2a). Furthermore, both plant architecture (GLM: χ^2_1_^ = 0.12, *p* = 0.73, Figure 3) and early exposure to HIPV had no effect on wasp landing preference (GLM: χ^2_1_^ = 1.66, *p* = 0.20, Figure 4).

**Figure 5:**
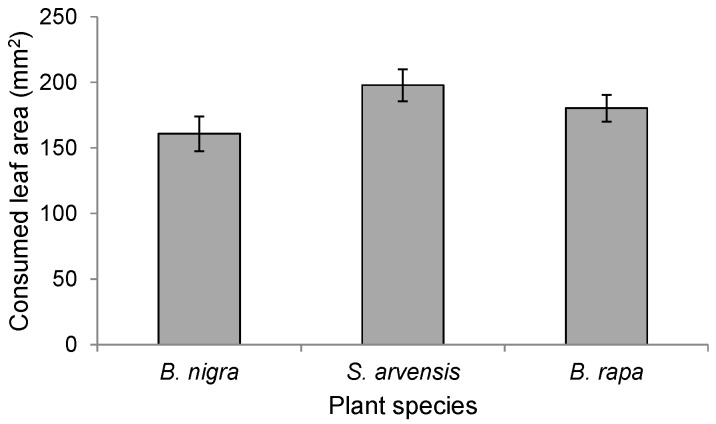
Leaf area consumed by 20 first instar *Pieris brassicae* feeding for 24 h on either *Brassica rapa*, *Sinapis arvensis*, or *Brassica nigra* plants. Bars present the means ± SE (*n* = 19).

There were significant differences in the amount of leaf tissues consumed by *P. brassicae* larvae among the three plant species (F_2,54_ = 3.38, *p* = 0.041) (Figure 5). The damage inflicted to *S. arvensis* plants was marginally, though not statistically, greater than the damage inflicted to *B. rapa* (Tukey test: *p* = 0.06, Figure 5) and *B. nigra* (Tukey test: *p* = 0.08, Figure 5). Damage levels were similar on *B. rapa* and *B. nigra* plants (Tukey test: *p* = 0.99, Figure 5).

### 2.3. Headspace Analysis

In the headspace of *B. rapa*, *S. arvensis*, and *B. nigra* that had been fed upon by *P. brassicae* larvae for 24 h, 33 different compounds were detected, of which 29 were present in the HIPV blend of all three host plant species (Table 1).

**Figure 6:**
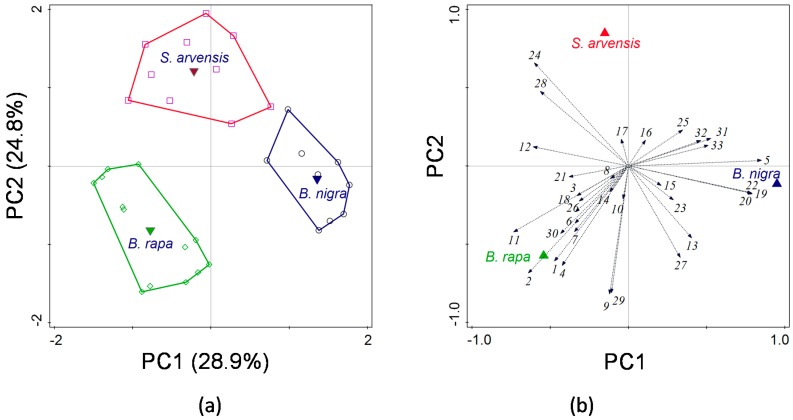
Principal component analysis (PCA) on the quantitative data of volatile compounds emitted by *Brassica rapa* (*n* = 13), *Sinapis arvensis* (*n* = 10), and *Brassica nigra* (*n* = 9) plants in response to *Pieris brassicae* feeding for 24 h. The score plots of the samples (**a**) depict separation of the different plant species along the first and second PC with the explained variance between brackets. The corresponding loading plot of the variables (**b**) shows the contribution of each volatile compound to the first two PCs and the sample groups (here plant species). For the identity of compounds presented as numbers in the loading plot (**b**), please refer to Table 1.

Based on PCA analysis of the volatiles, samples from the three plant species clearly separated (Figure 6a). The first PC, explaining 28.94% of the variation, separated *B. rapa* from *B. nigra*, whereas the second PC, explaining an additional 24.76% of the variation, further separated *S. arvensis* from *B. rapa* and *B. nigra* plants (Figure 6a). This means that the volatile blends emitted by *B. rapa* and *B. nigra* were more dissimilar compared to the blend emitted by *S. arvensis* plants. There was a significant difference in the total amount of volatiles emitted by the three plant species (F_2,29_ = 15.3, *p* < 0.001). *Brassica rapa* and *B. nigra* emitted a larger volume of volatiles than *S. arvensis* (Tukey multiple comparison tests: *B. rapa* vs. *S. arvensis* and *B. nigra* vs. *S. arvensis* both *p* < 0.05, *B. rapa* vs. *B. nigra p* > 0.05). Compounds that were emitted in higher amounts by *B. rapa* were the two nitriles: 2-methylbutanenitrile (ID 1), and 3-methyl-3-butenenitrile (ID 2); the glucosinolate hydrolysis product: 3-butenyl isothiocyanate (ID 9); the two green leaf volatiles (*Z*)-3-hexen-1-ol (ID 4) and (*Z*)-3-hexen-1-ol-acetate (ID 11) and the sesquiterpene (*E*,*E*)-α-farnesene (ID 29) (Figure 6b, Table 1). *B. nigra* plants were characterized by the relatively high emissions of the glucosinolate breakdown product allyl isothiocyanate (ID 5), and silphiperfolene isomers (ID 19, 20, 22), which were absent or only emitted in very small amount by the other two plant species (Figure 6b, Table 1). *S. arvensis* plants produced relatively more of the sesquiterpernes α-and β-caryophyllene (ID 24 and 28) (Figure 6b, Table 1).

## 3. Discussion

In this study, we show that rearing history only partially affected oviposition preference of *P. brassicae* butterflies and never resulted in a preference for the plant on which it had been feeding during larval development. It also had little or no effect on the foraging behaviour (i.e., plant preference) of its parasitoid, *C. glomerata*. Whereas *P. brassicae* butterflies reared on the different food plants did not exhibit any consistencies in oviposition preference behaviour, *C. glomerata* clearly preferred to alight on herbivore-damaged *B. rapa* plants. Preference of *C. glomerata* for *B. rapa* could not be explained by plant architecture, given that it is structurally similar, albeit slightly smaller, than the other two brassicaceous plant species studied here. Headspace analyses also revealed significant quantitative and qualitative differences among the HIPV blends emitted by the three plant species.

Natal experience has been reported to affect adult habitat selection by insects [27,33,34], but few studies have investigated this in lepidopteran species (butterflies and moths). Larval feeding experience with a feeding deterrent modified oviposition responses of subsequent adults in the moth species, *Ephestia cautella* and *Plodia interpunctella* [35] *Trichoplusia ni* [36], *Spodoptera littoralis* [37] and *Lobesia botran* [38]. To affect choices made during oviposition preference, cues obtained in the natal habitat must be memorized during larval feeding and carried through pupation to the adult stage. However, in holometabolous insects, the nervous system is profoundly reorganized during metamorphosis [39,40], which makes it unlikely that experience learned during the larval stage is easily retained in adult insects [25].

In nature, *P. brassicae* has up to three generations per year across much of its native range in Western and central Europe [31,41]. Furthermore, *P. brassicae* larvae require many food plants to support the successful development of a single brood, a requirement that limits the number of suitable plant species as oviposition sites in nature to about six or seven [32]. These plants, including the three species studied here, grow in large tightly assembled populations that enable the caterpillars to disperse from the natal plant to adjacent plants later during larval development by moving through the canopy [32,42]. Importantly, qualitatively and quantitatively suitable food plants are annuals or biennials with short growing seasons, and many of these plants also exhibit discrete periods of growth during the season. For example, *B. rapa* generally grows between March and May, *S. arvensis* between May and July, and *B. nigra* between June and August [31]. This means that different generations of *P. brassicae* must search for different host plants that generally grow at different locations, often a considerable distance away from the natal patch. Therefore, it is adaptive for *P. brassicae* that oviposition preference is not affected by larval rearing history; otherwise, the adults would risk wasting time searching for food plants that are no longer present (or which are no longer nutritionally suitable) in the natal habitat. Natal experience is only expected to affect plastic traits when it benefits animal fitness [29]. The fact *P. brassicae* is multivoltine and a specialist on short-lived, clustered brassicaceous plant species may explain why the effect of natal imprinting on adult oviposition preference is weak or non-existent. Though butterflies never preferred the plant on which they had been reared, the rank order of oviposition preference differed with natal experience; females preferred *B. rapa* plants for oviposition when reared on *B. oleracea* and *B. nigra* and preferred *B. nigra* when reared on *B. rapa* or *S. arvensis*.

Natal imprinting is not only found in lepidopteran species, but has also been observed in some species of Hymenoptera [28,43]. Several studies have shown that the response of adult female parasitoids to HIPV differs depending on the diet on which their host was feeding during larval parasitoid development inside of the host body [44,45]. For example, the ectoparasitoid *Hyssopus pallid* was more attracted to frass from its fruit-feeding host *Cydia pomonella* when the wasps had developed on hosts fed on apples compared to wasps reared on hosts fed on artificial diet [46]. The length of rearing history can also play a role in the wasp’s future plant volatile preferences [47]. For instance, when *Plutella xylostella* that are parasitized by *Diadegma semiclausum* were fed on snow pea for three successive generations, female wasps showed a relatively higher preference to snow pea volatiles in the third than in the first generation [47]. In our study, the parasitoid was only reared on hosts and plants for a single generation, reflecting conditions found in nature, where different generations generally must find hosts on different plant species. Consequently, we found that natal experience had no effect on volatile-mediated foraging behaviour in *C. glomerata*. The adaptive potential of natal imprinting clearly depends on such factors as the reliability of being associated with the same plant species or the degree of chemical and structural similarity of different plant species that may be used in successive generations by the herbivore and its parasitoid. Natal experience is adaptive when the environment is predictable over several generations in an insect. For multivoltine parasitic wasps, where different generations also need to search for hosts on different plant species in different habitats, it is important that natal experience exerts little effect on their landing preference.

We also found that early exposure to HIPV at eclosion had no effect on wasp landing preference. By contrast, host plant stimuli have been reported to increase a parasitoid’s attraction to the natal host plant [48]. For example, the attraction of the parasitoid wasp *Trichogramma brassicae* to tomato plants increased when the wasps were allowed to emerge from their hosts in the presence of these plants. Similarly, attraction of *C. congregata* to cherry volatiles increased when the parasitoid had physical contact with the host plant at eclosion. Therefore, the importance of conditioning at eclosion appears to be association-specific and even differs amongst closely related taxa (e.g., *C. glomerata* and *C. congregata*). This could be due to differences in life-history traits among the plants and insects. In the case of *C. congregata*, its herbivore hosts (e.g., the larvae of sphingid moths) may associate with the same food plants and/or habitats over successive generations, making conditioning adaptive. Certainly more plant host–parasitoid associations need to be studied to extrapolate potential relationships between the life-history of the plants and hosts and conditioning/innate responses in their parasitoids.

Female parasitoids clearly preferred *B. rapa* over the other two cruciferous species. Several factors could contribute to this preference. *B. rapa* grows early in the season and may therefore be one of the few plant species available in the Netherlands for *P. brassicae* when they emerge from winter diapause. Consequently *C. glomerata* may have evolved a strong sensitivity to (volatile) cues related to the first available food plant of its host. Alternatively, some of the volatiles emitted by *B. rapa* may trigger a stronger sensory response in the parasitoid than compounds in the blend of *B. nigra* and *S. arvensis*. Little is still known as to the identity of specific volatiles or volatile blends that are most attractive to parasitoids [45], although some compounds have been shown to play an important role in enhancing attractiveness of the blend [49,50].

The analysis of the HIPV blends showed that there were significant quantitative and qualitative differences among the three plant species. The total amounts of volatiles from *B. rapa* and *B. nigra* were significantly larger than from *S. arvensis*. When HIPV blends induced by different treatments of the same plant species are compared, quantitative aspects of these blends may to a large extent determine parasitoid attraction [49,51]. However, when parasitoid attractiveness to HIPV blends emitted by different plant species is compared, qualitative rather than quantitative aspects may be more important [45]. It is known that blends produced by species in the Brassicaceae vary dramatically across different species [52,53].

All brassicaceous plant species produce glucosinolates [54], which function as defensive compounds against a range of attackers such as pathogens and insect herbivores [20,55]. Deterrent or toxic activity only emerges after tissue damage, e.g., by caterpillar feeding and concomitant release of the enzyme myrosinase, which is stored in specialized cells. This enzyme catalyses the conversion of glucosinolates into toxic hydrolysis products, of which many are volatile [55]. These volatile breakdown products have been shown to serve as reliable signals for the parasitoid *C. rubecula* to their host, *P. rapae* [56]. If breakdown products of glucosinolates play a role in host plant selection by *C. glomerata*, the high amounts of 3-butenyl isothiocyanate, which is the breakdown product of gluconapin, the dominant glucosinolate in *B. rapa*, may explain its enhanced attraction to this plant. However, this does not explain why *B. rapa* is more attractive than *B. nigra* which emits allyl isothiocyanate in even larger amounts than *B. rapa* emits 3-butenyl isothiocyanate (allyl isothiocyanate is a hydrolysis product of sinigrin, the dominant glucosinolate in *B. nigra*). *Diaeretiella rapae*, a parasitoid of the aphid *Brevicoryene brassicae* that is a specialist of brassicaceous plants, was shown to be more attracted to synthetic 3-butenyl isothiocyanate than to 4-pentenyl isothiocyanate [50], although it is also attracted to synthetic allyl isocyanate [57]. This suggests that isothiocyanates are differentially attractive to parasitoids. The low volatility of hydrolysis products of sinabin, the dominant glucosinolate in *S. arvensis*, may be responsible for the absence of these compounds in the headspace of *S. arvensis* explaining the reduced attractiveness of these plants to *C. glomerata*.

In summary, our study reveals that rearing history has little or no effect on oviposition preference of *P. brassicae* butterflies or landing preference of its major parasitoid *C. glomerata*. Oviposition preference of *P. brassicae* shifted between *B. nigra* and *B. rapa*, but the butterflies never displayed a clear preference for the plant species on which they had been reared. *C. glomerata* had a clear preference for host-infested *B. rapa* plants. For multivoltine insects, such as *P. brassicae* and *C. glomerata* that primarily rely on short-lived annuals for immature development, it is a challenge for different generations to locate suitable host plants, given that they are forced to leave the natal habitat to do so. Therefore, it is adaptive that these insects are labile in the cues that they use in host plant location behaviour and, thus, that it is not affected by natal imprinting. Furthermore, our study also shows that the herbivore and the parasitoid use different cues when searching for food or host plants. To better understand the mechanisms that underline these interactions, it is important to examine an array of ecophysiological constraints on the insects and the traits the insects exhibit to counter them. Clearly, the biology and phenology of the food plant(s) leave an indelible mark on their insects.

## 4. Materials and Methods

### 4.1. Plants

*B. rapa*, *B. nigra* and *S. arvensis* seeds were collected from natural growing populations in Gelderland, The Netherlands. Seeds were germinated and seedlings were subsequently transferred to 1.1-L pots filled with peat soil (Lentse potgrond no.4; lent, The Netherlands). Plants were grown in a greenhouse at the Netherlands Institute of Ecology (NIOO) under the following conditions: 21 ± 2 °C (day) and 16 ± 2 °C (night), 50% relative humidity, and a photoperiod of at least 16 h. The plants were watered twice a week during the first 3 weeks of development. When the plants were 3 weeks old, they were fertilized once a week with Hoagland solution, which was applied to the soil. Watering and fertilization continued during the experiments.

As the insects have been reared on Brussels sprout plants, *Brassica oleracea* L. var. gemmifera cv. Cyrus for many (>10) generations, this plant was used as a control. Brussels sprout plants were grown from seeds in peat soil in 1.1-L plastic pots in a greenhouse (50%–70% relative humidity, 20–25 °C, and a photoperiod of 16 h) and were 4 to 5 weeks old when used in the experiments.

### 4.2. Insects

*P. brassicae* and *C. glomerata* were collected in experimental fields near Wageningen, The Netherlands. *P. brassicae* caterpillars were reared on Brussels sprout plants in a greenhouse at 50%–70% relative humidity, 20–25 °C, and a photoperiod of 16 h at Wageningen University (WU). *C. glomerata* was reared on young *P. brassicae* caterpillars feeding on Brussels sprouts. Once the fully developed larvae of *C. glomerata* emerged from *P. brassicae* hosts and had spun cocoons, they were collected for further rearing or experimental purposes. Approximately five days after cocoon formation, adult wasps emerged at which point they were provided with 10% sugar solution.

### 4.3. Preparation of Insects Used in Experiments

#### 4.3.1. Herbivore

*P. brassicae* were reared from egg-to-adult for one generation on one of the three host plant species: *B. rapa*, *S. arvensis* or *B. nigra*. We also determined oviposition preference of butterflies reared on *B. oleracea* on which they had been reared for many generations. Single four-week-old *B. oleracea* and three-week-old *B. rapa*, *S. arvensis*, or *B. nigra* plants were placed in the rearing cage with adult *P. brassicae* butterflies for 24 h. Plants with egg clusters were transferred to a cage with additional plants of the same species as the one on which the eggs were laid. Eggs were allowed to develop into pupae on their respective food plants. Eclosing butterflies were provided with a (20%) honey solution and were allowed to mate. Butterflies were 3–5 days old when they were used in the choice bioassays.

#### 4.3.2. Parasitoid

*C. glomerata* were reared for one generation on *P. brassicae* caterpillars feeding on one of the four host plant species: *B. oleracea*, *B. rapa*, *S. arvensis* or *B. nigra*. Caterpillars of *P. brassicae* were obtained and reared as described in the previous section until they reached the mid first instar stage. For parasitism, female wasps were collected from the general culture. First instar *P. brassicae* caterpillars were parasitized by presenting them individually to a female wasp. After parasitism by *C. glomerata*, caterpillars were introduced onto one of the four host plant species (*B. oleracea*, *B. rapa*, *S. arvensis* or *B. nigra*), which were maintained in separate cages until the larvae of the parasitoids emerged and formed cocoons. Parasitoid cocoons were collected in Petri dishes (9.5 cm) and were maintained in an incubator at 21 ± 1 °C until adult eclosion at which point they were transferred into 30 × 30 × 30 cm (Bugdorm) plastic cages and provided with 10% sugar, water, and honey. Female wasps used in the bioassays were 2–8 days old.

### 4.4. Host-Plant Oviposition Preference of Pieris brassicae Butterflies

Oviposition preferences were assessed in three-choice experiments in six outdoor tents (3 × 4 × 2 m) placed on bare soil in an experimental field adjacent to WU. Plants from each species were prepared as described in the *Plants* section.

Single plants of each of the three plant species were randomly placed in a triangle, approximately 1.5 m apart, in the experimental tents. One female and one male butterfly were released in the middle of the tent. A bioassay was terminated and its choice recorded when a female butterfly had laid the first egg clutch, which was checked three times a day. Females were used only once. The bioassay was repeated at least 30 times with butterflies being reared on the same plant species. Bioassays were conducted from June to August 2013. New plants were used for each replicate and the positioning of the plant species in the tent was randomized.

### 4.5. Host-Plant Landing Preference of the Parasitoid, Cotesia glomerata

In a wind tunnel set-up (see below), we determined HIPV mediated landing preference of female *C. glomerata* parasitoids when reared for one generation from *P. brassicae* caterpillars developing on of the host plants, *B. rapa*, *S. arvensis* and *B. nigra*, respectively. In addition, we used wasps that had developed in *P. brassicae* feeding on *B. oleracea*, the food plant on which the insects had been reared for >10 generations. In the wind tunnel, plant pairs, i.e., all three combinations of host-infested *B. rapa*, *S. arvensis* and *B. nigra* plants were offered to parasitoids reared on the four different food plants. Individual plants were infested with 20 first instar *P. brassicae* or 10 second instar *P. brassicae* caterpillars, depending on caterpillar availability, and incubated in a greenhouse for 24 h at 50%–70% relative humidity and 20–25 °C with a photoperiod L:D of 16:8 h. Plant combinations used in single choice bioassays were always infested with the same number of caterpillars of the same instar.

To determine whether differences in the amount of feeding damage affected landing preference, we determined for each of the three plant species (*n* = 19 per plant species) the amount of leaf tissue consumed from plants infested by 20 first-instar *P. brassicae* larvae for 24 h. Damaged areas were calculated using millimeter paper on transparent plastic sheets.

The plant species differ in their architecture, which could affect landing preference of the wasps. For instance, *S. arvensis* and *B. nigra* grow taller than *B. rapa*, which has a shorter main stem and leaves that initially expand horizontally. In an additional wind tunnel experiment, we examined the architectural influence on HIPV preference using single detached leaves from *B. nigra* and *B. rapa* instead of intact plants. The wasps used in this experiment had been reared from *P. brassicae* larvae on *B. nigra*. Leaves infested by 20 first-instar *P. brassicae* larvae for 24 h were cut and put into vials with water, and were allowed to recover for 2–4 h before they were used in a wind tunnel experiment. This comparison was tested in 10 replicate bioassays.

Furthermore, wasps can also be conditioned by exposure to HIPVs when they emerge from the host caterpillars prior to pupation and cocoon construction but in the presence of plant material [58]. As described above, one group of wasps was collected and separated from its host and the host plants prior to egression and cocoon construction, whereas another group of wasps was left with its host and host plants through egression and cocoon construction until adult eclosion. Landing preference (*n* = 10) was compared when wasps of these two groups were offered a host-infested *S. arvensis* and *B. rapa* plant, while the insects had been reared on *S. arvensis*.

### 4.6. Wind Tunnel Experiment

Volatile-mediated foraging behaviour was studied in a wind tunnel set-up, which is described in detail in [59]. The environmental conditions were set as follows: wind speed, 0.1 m·s^−1^; light intensity 500–1000 Lux: temperature 25 ± 1 °C; relative humidity 60% ± 5%. To stimulate foraging of *C. glomerata*, females were exposed to a host-damaged Brussels sprout leaf from which the *P. brassicae* caterpillars had been removed. Female wasps were collected in 7-mL glass vials and wasps were released individually in a “release cylinder” located in the middle of the wind tunnel. Two test plants were placed approximately 60–70 cm up-wind from the release cylinder.

Each wasp was observed for a maximum of 15 min. When a wasp did not land on one of the two plants within 15 min, it was recorded as “non-responding” and this data point was excluded from the statistical analysis. In each bioassay with one test plant combination, we tested 10 responding wasps, which served as a single data point. Each test plant combination in relation to the wasp’s rearing history was tested 8–10 times with a new set of plants and each wasp was used only once. The response of a total of 1170 wasps was recorded in the bioassays examining the effect of rearing history, whereas 100 wasps were tested in each of the two additional bioassays.

### 4.7. Volatile Collection and Analysis of Herbivore Infested Plants

Volatiles emitted by *B. rapa*, *S. arvensis*, and *B. nigra*, which had been exposed to feeding by 20 first instar *P. brassicae* caterpillars for 24 h were collected and analysed. Plants were treated similarly as described for the behaviour bioassay. Volatiles were collected from individual plants, with 9–13 plants per species. The potting soil of the plants around the stem was wrapped in aluminium foil to reduce the release of plastic- and soil-related volatiles before the plants with the caterpillars remaining on them were transferred to a 30-L glass jar containers. Glass jars were sealed with viton-lined glass lids equipped with an air inlet and outlet. Pre-cleaned compressed air filtered through charcoal was led into the glass jars, and the plants were allowed to acclimatize for 40 min. Dynamic headspace volatile collection was carried out in a laboratory at 20 ± 2 °C, by sucking air out of the jar at a rate of 200 mL·min^−1^ for 2 h through a stainless steel cartridge containing 200 mg Tenax TA (20/35 mesh; CAMSCO, Houston, TX, USA). Immediately after volatile collection, foliar fresh weight of each plant was measured and the Tenax TA cartridges containing sample volatiles were dry-purged under a flow of nitrogen (50 mL·min^−^^1^) for 10 min at room temperature (21 ± 2 °C) to remove moisture and stored till analysis. Periodically, volatiles from just pots with soil wrapped in aluminium foil were collected and the compounds recorded, together with the volatiles originating from the Tenax TA adsorbent and the analytical instruments were excluded as artefacts from the data obtained for the plant samples as a correcting measure. Headspace volatile samples were analysed by using a Thermo Trace Ultra Gas Chromatography (GC) coupled to a Thermo Trace DSQ quadrupol mass spectrometer (MS) (both from Thermo (Thermo Fisher Scientific, Waltham, MA, USA) and were used for the separation and detection of volatile compounds. For details of the analytical protocol please refer to [60].

### 4.8. Statistical Analysis

To statistically analyse *P. brassicae* female butterfly oviposition preference, we used χ^2^-tests comparing the observed oviposition preference counts for the three plant species with an expected distribution of 1:1:1. When the test result was significant, we conducted pairwise χ^2^-square tests with α = 0.05/3 to correct for type I errors (Bonferroni correction).

The response variable in the statistical analyses of the *C. glomerata* wind tunnel bioassays is the fraction of wasps out of the total of 10 responding wasps choosing one of the plant species that was set to be the focal odour source. We used logistic regression, i.e., a generalized linear model (GLM) with binomial variable distribution for errors and a logit link function to determine the effect of rearing history on wasp landing preference for each plant pair combination. In the case of over-dispersion, we corrected for this by allowing the variance functions of the binomial distribution to have a multiplicative over-dispersion factor. Plant species on which the wasps were reared was entered as the explanatory variable in the regression model. To determine whether there was a significant preference for one of the odour sources within a plant pair, we tested H_0_ = logit = 0 based on model term estimates of the GLM model. Additionally, we used one-sample *t*-tests to determine whether there was a preference for one of the two odour sources within a plant-pair comparison ignoring the effect of diet with H_0_: no preference, mean preference fraction is 0.5. We used a similar GLM approach for the data on the effect of plant architecture and the effect of early HIPV exposure on wasps landing preference, respectively.

We used one-way ANOVA to examine differences in leaf damage on the three host plants.

A multivariate statistical approach was used, i.e., principal component analysis (PCA), to visualize whether volatile profiles could be separated according to plant species and to determine which volatile compounds contributed the most to the separation. All volatiles compounds were included in the analysis. Univariate Kruskal–Wallis tests were employed to reveal significant differences in the emission of each volatile among the three plant species. We used ANOVA to analyse differences in total amounts of volatiles (log-transformed).

PCA analysis on all volatile data was performed in Canoco version 5.03 (ter Braak and Šmilauer, Microcomputer Power, Ithaca, NY, USA). All the other analyses were performed using SAS 9.3 (SAS Institute Inc., Cary, NC, USA).

## 5. Conclusions

We have shown that, in an insect herbivore and its gregarious endoparasitoid, innate, conditioned responses are unimportant in terms of food plant preference. This is adaptive because of life-history traits in the herbivore and its parasitoid: the food plants are all short-lived annuals with little temporal overlap in seasonal phenology and the insects are multivoltine, with up to three generations per year. Therefore, in nature, different generations of the insects are obligatorily associated with different plant species that may grow some distance apart.

## Figures and Tables

**Table 1:** Volatile compounds emitted by *Brassica rapa*, *Sinapis arvensis*, and *Brassica nigra* plants damaged by 20 first instar *Pieris brassicae* caterpillars for 24 h.

ID ^b^	Plant Species	*B. rapa* ^a^	*S. arvensis*	*B. nigra*
	Compound	(*n* = 13)	(*n* = 10)	(*n* = 9)
1	2-Methylbutanenitrile ***	91.8 ± 24.4	3.7 ± 0.8	3.8 ± 0.4
2	3-Methyl-3-butenenitrile ***	6.1 ± 2.0	ND	ND
3	(*E*)-2-Hexenal	0.7 ± 0.1	0.6 ± 0.2	0.3 ± 0.07 ^7,!^
4	(*Z*)-3-Hexen-1-ol ***	20.3 ± 3.0	6.7 ± 1.4	7.5 ± 2.0
5	Allyl isothiocyanate ***	0.2 ± 0.1 ^7^	1.4 ± 0.6 ^7^	135.2 ± 22.6
6	Butane, 1-isothiocyanato ***	14.7 ± 5.3	0.5 ± 0.2 ^8^	0.5 ± 0.08 ^8^
7	(*E*)-4-Oxo-2-hexenal *	16.5 ± 2.7	14.9 ± 6.5 ^8^	5.6 ± 2.4 ^7^
8	Sabinene	0.9 ± 0.1	1.1 ± 0.2 ^9^	1.0 ± 0.1
9	3-Butenyl isothiocyanate ***	20.1 ± 5.1	0.05 ± 0.08 ^2^	0.7 ± 0.1
10	Myrcene	3.5 ± 0.3	2.9 ± 0.4	3.0 ± 0.3
11	(*Z*)-3-Hexen-1-ol, acetate	165.4 ± 20.8	49.7 ± 9.4	21.9 ± 7.9
12	Hexanoic acid, 2-ethyl-, methyl ester ***	1.6 ± 1.2	3.6 ± 2.7	0.1 ± 0.09 ^6^
13	(*E*)-DMNT *	26.4 ± 11.0	4.3 ± 1.2	28.8 ± 7.7
14	Unknown	0.4 ± 0.02	0.3 ± 0.07 ^8^	0.4 ± 0.04
15	Menthol	2.7 ± 0.9	2.0 ± 0.3	3.7 ± 0.8
16	Unknown	0.5 ± 0.1	1.1 ± 0.3	0.8 ± 0.3
17	Unknown	0.7 ± 0.2	1.6 ± 0.6	0.8 ± 0.3
18	Methyl salicylate ***	2.6 ± 0.7	0.3 ± 0.04	0.7 ± 0.2 ^8^
19	Presilphiperfol-7-ene ***	ND	ND	0.4 ± 0.1 ^7^
20	7-β-H-Silphiperfol-5-ene ***	ND	ND	0.5 ± 0.2 ^7^
21	α-Terpinyl acetate *	0.3 ± 0.05	0.4 ± 0.06 ^9^	0.2 ± 0.06 ^7^
22	Silphiperfol-6-ene ***	ND	ND	0.2 ± 0.1 ^7^
23	α-Funebrene **	0.1 ± 0.05 ^11^	0.09 ± 0.02 ^8^	0.6 ± 0.1
24	β-Caryophyllene **	2.2 ± 1.4 ^6^	9.3 ± 3.7	0.02 ± 0.05 ^1^
25	(*E*)-α-Bergamotene	0.09 ± 0.06 ^6^	0.1 ± 0.04 ^7^	0.2 ± 0.08 ^7^
26	α-Guaiene	0.1 ± 0.03	0.1 ± 0.03 ^8^	0.09 ± 0.03 ^8^
27	(*E*)-β-Bergamotene	0.2 ± 0.2 ^5^	0.05 ± 0.2 ^1^	0.2 ± 0.1 ^5^
28	α-Caryophyllene	0.5 ± 0.3 ^5^	1.1 ± 0.6 ^6^	0.03 ± 0.08 ^1^
29	(*E*,*E*)-α-Farnesene ***	22.8 ± 10.6	0.3 ± 0.2 ^4^	0.9 ± 0.3 ^7^
30	α-Bulnesene	0.1 ± 0.04 ^8^	0.06 ± 0.1 ^2^	0.04 ± 0.1 ^1^
31	Methyl *cis*-dihydrojasmonate	7.1 ± 1.3	9.5 ± 1.3	12.0 ± 1.1
32	Unknown *	20.0 ± 2.7	30.0 ± 6.7	43.1 ± 6.3
33	Unknown *	4.7 ± 0.6	6.8 ± 1.3	10.0 ± 1.3
	Total ***	433.5 ± 50.4	152.4 ± 25.9	283.2 ± 32.7

^a^ Volatile emissions are given as a mean peak area ± SE/g fresh weight of foliage divided by 10^5^ with number of sample replicates (*n*) between brackets; ^b^ ID corresponds with the number presented in loading plot (Figure 6b); ^!^ Numbers in superscript following emission quantities represent the number of samples in which a given compound was detected. ND = not detected in any of the samples; * Compounds with asterisks indicate significant differences in emission quantities of volatiles among the three plant species (Kruskal-Wallis one-way ANOVA, * *p* < 0.05; ** *p* < 0.01; *** *p* < 0.001); (*E*)-DMNT: (*E*)-4,8-dimethylnona-1,3,7-triene.
